# Optimization of an economical medium composition for the coculture of *Clostridium butyricum* and *Bacillus coagulans*

**DOI:** 10.1186/s13568-022-01354-5

**Published:** 2022-02-15

**Authors:** Yonghong Li, Yun Wang, Yingying Liu, Xuan Li, Lifei Feng, Keke Li

**Affiliations:** 1 Key Laboratory of Advanced Drug Preparation Technologies, Ministry of Education, School of Pharmaceutical Sciences, Zhengzhou University, Zhengzhou, 450001 Henan China; 2 Henan Province Collaborative Innovation Center of New Drug Research and Safety Evaluation, Zhengzhou, 456150 Henan China; 3 HeNanJinBaiHe Biotechnology Co., LTD, Anyang, 450000 Henan China

**Keywords:** Optimization, Medium composition, Co-culture, *Clostridium butyricum*, *Bacillus coagulans*

## Abstract

*Clostridium butyricum* is a spore-forming probiotic which can promote the enhancement of beneficial bacteria and maintain intestinal microecological balance. However, it is difficult to improve the production level of *C. butyricum* by conventional fermentation process. In this study, a co-fermentation process of *C. butyricum* DL-1 and *Bacillus coagulans* ZC2-1 was established to improve the viable counts and spore yield of *C. butyricum*, and the formula of coculture medium was optimized by flask fermentation. The results showed that the optimum medium composition is 10 g/L bran, 15 g/L corn steep powder, 15 g/L peptone, 1 g/L K_2_HPO_4_ and 0.5 g/L MnSO_4_.Cultured stationarily in the optimal medium for 36 h, the number of viable bacteria of *C. butyricum* DL-1 reached 1.5 × 10^8^ cfu/mL, Which was 375 times higher than that incubated in the initial medium. The sporulation rate reach 92.6%. The results revealed an economical and effective medium composition for the coculture of *C. butyricum* and *B. coagulans*, which achieved a 64.6% cost reduction. The co-fermentation process established in this study provides a new fermentation mode for the industrial production of other absolute anerobic bacteria.

**Supplementary Information:**

The online version contains supplementary material available at 10.1186/s13568-022-01354-5.

## Key points

- A novel co-culture system was established.
- Medium composition for the coculture of *C. butyricum* and *B. coagulans* was optimized.
- The optimal medium cost is lower than what was reported.

**Supplementary Information:**

The online version contains supplementary material available at 10.1186/s13568-022-01354-5.

## Introduction

Probiotics can improve human health (Li et al. 2020a). They are effective in enhancing intestinal immunity (La Fata et al. 2018) and treating diseases such as dyslipidemia (Matey-Hernandez 2017), non-alcoholic high-fat diet-induced liver disease (Liu et al. 2017) and cancers (Jones et al. 2013). Increasing numbers of probiotics have been used as medicines and feed additives recently. Commonly used probiotics species include yeast (Nelson et al. 2020), *Bacillus* (Mingmongkolchai and Panbangred 2018), *lactobacillus* (Das et al. 2020) and *Bifidobacterium* (Tian et al. 2020). *C. butyricum* (Poolsawat et al. 2019) is a promising emerging member.

*C. butyricum*, a strictly anaerobic spore-forming probiotic (Li et al. 2020b), can regulate the imbalance of host intestinal flora and maintain microecological balance (Cassir et al. 2016; Kanai et al. 2015; Pan et al. 2019). It antagonizes pathogenic bacteria and promotes the proliferation of intestinal beneficial bacteria (Li et al. 2016, 2019b). Therefore, it is widely used to improve human immunity and treat intestinal flora imbalance (Hai-dong Lia 2018). It is also used in animal husbandry to improve animal production performance (Khajeh Bami et al. 2020). With the complete ban of antibiotics as feed additives, *C. butyricum* has a promising market prospect as an antibiotic substitute because of its marked animal health protection performance (Yi et al. 2020). Additionally, *C. butyricum* and *B. coagulans* can effectively inhibit *Helicobacter pylori* with few adverse events (Zhang et al. 2020), and could be used as alternative anti-Helicobacter pylori drug. They were also used in clinical treatment for acute Enteritidis and Intestinal microbiologic disorder.

As an obligate anaerobe, *C. butyricum* need strict anaerobic condition. Even provided with absolute anaerobic condition, the viable counts and spore yield of *C. butyricum* was not as high as that of other probiotics. The spore yield of *C. butyricum* is usually less than 1 × 10^9^ cfu/mL in industrial production.

While the spore yield of *B. subtilis* reached 8.78 × 10^9^ cfu/mL after the optimization of medium components and culture conditions (Posada-Uribe et al. 2015). And the viable counts of *L. plantarum* and *L. paracasei* reached 2.77 × 10^9^ and 2.78 × 10^9^ cfu/g in anaerobic solid-state co-fermentation, respectively (Chen et al. 2020).

Solid-state co-fermentation of *C. butyricum* and other probiotics can effectively enhance *C. butyricum* growth and sporulation (Su et al. 2018), suggesting that co-fermentation may be an effective alternative for obligate anaerobes. Mixed fermentation creates a biological hybrid system (Englezos et al. 2019), in which microorganisms synergistically metabolize and establish mutually beneficial symbiosis, providing better fermentation condition than their purebred counterpart (Hamid et al. 2019). Co-fermentation are widely used in food and feed industry. Dromedary yogurt was fermented with *L. bulgaricus* and *Streptococcus thermophiles* to improve its nutrition, texture and syneresis (Jrad et al. 2020). *Rhizopus oligosporus* and *L. plantarum* co-fermentation is an effective method to increase the antioxidant potential of Grass pea and flaxseed oil-cake (Stodolak et al. 2020).

In this study, a co-culture system of *C. butyricum* DL-1 and *B. coagulans* ZC2-1 were established. In the co-fermentation system, the facultative anaerobic *B. coagulans* strain consumes oxygen in the culture medium and provides anaerobic environment for the absolute anaerobic *C. butyricum* strain. The culture medium composition of co-fermentation process was optimized so as to obtain high viable counts and spore yield of *C. butyricum* at low medium cost. The co-fermentation process provided a new energy-saving fermentation mode for other absolute anaerobic microbes.

## Materials and methods

### Inoculum

*C. butyricum* DL-1 strain was provided by Jinbaihe biotechnology Co, Ltd in Tangyin Country, Anyang City, Henan Province, China. Its 16S rRNA gene sequences have been deposited in GenBank with the accession numbers of MW218001.

### Medias

The broth media contained 10 g/L peptone, 3 g/L beef extract, and 5 g/L NaCl, with a pH value of 7.0 ± 0.1. Solid nutrient agar media composed of 10 g/L peptone, 3 g/L beef extract, 5 g/L NaCl, and 10 g/L agar with a pH value of 7.0 ± 0.1. Solid acid-producing bacteria selection media contained 5 g/L glucose, 5 g/L peptone, 1 g/L yeast extract powder, 0.3 g/L CaCO_3_, and 10 g/L agar with a pH value of 7.0. Proliferation medium of *C. butyricum* DL-1 was composed of 5 g/L glucose, 5 g/L sodium chloride, 3 g/L sodium acetate trihydrate, 10 g/L tryptone, 3 g/L yeast extract, 10 g/L beef extract, 1 g/L soluble starch, 0.5 g/L L-cysteine hydrochloride, pH 7.2. Proliferation medium of *B. coagulans* was composed of 10 g/L glucose, 5 g/L yeast extract, 10 g/L peptone, pH 6.8–7.0. Initial coculture media of *C. butyricum* DL-1 and the four *B. coagulans* strains was composed of 10 g/L glucose, 10 g/L tryptone, 5 g/L yeast extract, pH 6.8–7.0, all the mediums were sterilized at 121 °C for 20 min.

### *B. coagulans* acclimation and screening

Inoculum samples were taken from the gut of health chicken and commercial kimchi. The samples diluted by 10 times with sterile water were shaken for 30 min, and then let stand for 10 min. The supernatants obtained were water bathed at 80 °C for 20 min and then inoculated to the broth medium. Then, the mixture was cultured at 200 rpm and 37 °C for 24 h. Culture broth of appropriate concentration was spread on the solid acid-producing bacteria selection media and cultured at 37 °C stationarily for 48 h. The colonies with obvious calcium-dissolving circles were streaked on solid nutrient agar plates for further purification. The pure colonies were preserved on nutrient agar slants at 4 °C for further study.

The screened strains were identified by morphological characterization combined with phylogenetic analysis. Microbial phenotypic characteristics were determined by observing colonial and mycelia morphology by naked eyes and an optical microscope, respectively. Phylogenetic analysis was based on their 16S ribosomal DNA sequences. The 16S rRNA gene sequence fragments were amplified with the primer pair of 27F (5′-AGAGTTTGATCCTGGCTCAG-3′) and 1492R (5′-GGTTACCTTGTTACGAC T T-3′). The PCR protocol consisted of the following steps: 5 min at 94 °C for the first denaturation step, followed by 30 cycles of denaturation at 94 °C for 30 s, annealing at 55 °C for 30 s, and extension at 72 °C for 2 min, and ended with a final extension step at 72 °C for 7 min. The PCR reaction mixture (20 μL) consisted of an appropriate amount of DNA template (10–100 ng), 1.0 μL of Taq DNA polymerase (Beijing Com Win Biotech Co. Ltd., China), 0.5 μL of 10.0 μM each primer, and 8.5 μL of ddH_2_O. The reaction mixture without template DNA was used as a negative control. The PCR product was verified using agarose gel electrophoresis and purified using the QIA Quick purification kit. Pure PCR products were sequenced by TsingKe Biological Technology Co. Ltd., Zhengzhou, China.

Multiple sequence alignments were performed with ClustalW in MEGA 6, and phylogenetic trees were constructed from the evolutionary distance data calculated from Kimura’s two-parameter model using the neighbor-joining method by MEGA 6. Bootstrap analyses were performed based on 1000 random resampling. Reference sequences were retrieved from GenBank with the accession numbers indicated in the trees.

### Selection of coculture system

*B. coagulans* inoculum was cultured in proliferation medium at 37 °C and 180 rpm for 16 h. *C. butyricum* DL-1 inoculum was cultured at 37 °C stationarily for 16 h, with the shake flasks sealed with 8 layers of gauze and 2 layers of kraft paper. The co-fermentation condition was as follows: loading liquid ratio was 40%, inoculation ratio of *C. butyricum*DL-1 and *B. coagulans* strains was 6% and 4%, respectively. The shake flask was sealed with 8 layers of gauze and 2 layers of kraft paper were cultured stationarily at 37 °C. After a 24 h culture, the viable counts and spore concentration were analyzed.

### Determination of total viable counts and spore yield of *C. butyricum* DL-1

After sequential tenfold dilution of cell suspensions of *C. butyricum*, 100 µL of samples were spread on agar plates. The colonies formed after incubation at 37 °C for 16 h were counted and statistically analyzed. Viable counts were expressed as colony-forming units per milliliter (cfu/mL). Spore concentration was measured by the same method as that of viable counts except that the cell suspension was heated at 80 °C for 10 min in advance. Spore yield was calculated as the percentage of spore count to total viable cells of *C. butyrium* DL-1. Three replicates were set for each dilution.

### Growth curve of strain *C. butyricum *DL-1 and *B. coagulans* ZC2-1

Specific *C. butyricum* DL-1 proliferation medium inoculated at a 4% inoculum size was cultured stationarily at 37 °C, with a 60% filling volume. OD_600_ and pH of the culture broth were assayed every 4 h. Growth process of *B. coagulans* ZC2-1 was monitored by the same protocol as that of *C. butyricum*DL-1, except that the inoculated proliferation medium was cultured at 180 rpm, with a filling volume of 20%.

### Optimization of co-fermentation medium formula of strain *C. butyricum*DL-1 and *B. coagulans* ZC2-1

Single-factor tests were used to study the effect of carbon source, nitrogen source, and inorganic salts on the viable counts and spore yield of *C. butyricum*DL-1. The factors were studied successively, and the optimization results were used in subsequent experiment step. Bran, corn starch, soluble starch, sucrose, lactose, maltose and glucose were used as alternative carbon source. Corn steep powder, soybean meal powder, peptone, beef extract, fishmeal, tryptone, yeast extract and yeast were used as alternative nitrogen source. NaCl, KCl, K_2_HPO_4_, CaCO_3_, MnSO_4_, MgSO_4_, and Sodium acetate trihydrate [C_2_H_3_NaO_2_·(H_2_O)] were used as candidate inorganic salts. Viable counts and spore yield of *C. butyricum* DL-1 were analyzed at regular intervals with the methods described above. Three parallel experiments were conducted for each experimental group.

Based on the results of single-factor tests, a L_9_(3^3^) orthogonal experiment was designed to optimize the concentration of carbon source and nitrogen source, and a L_9_(3^2^) orthogonal experiment was designed to study the effect of inorganic salts concentration on the viable counts and spore yield of *C. butyricum* DL-1. The orthogonal experiment was designed by SPSS 20.0 software.

## Results

### Isolation and identification of *B. coagulans* strains

**Fig.1:**
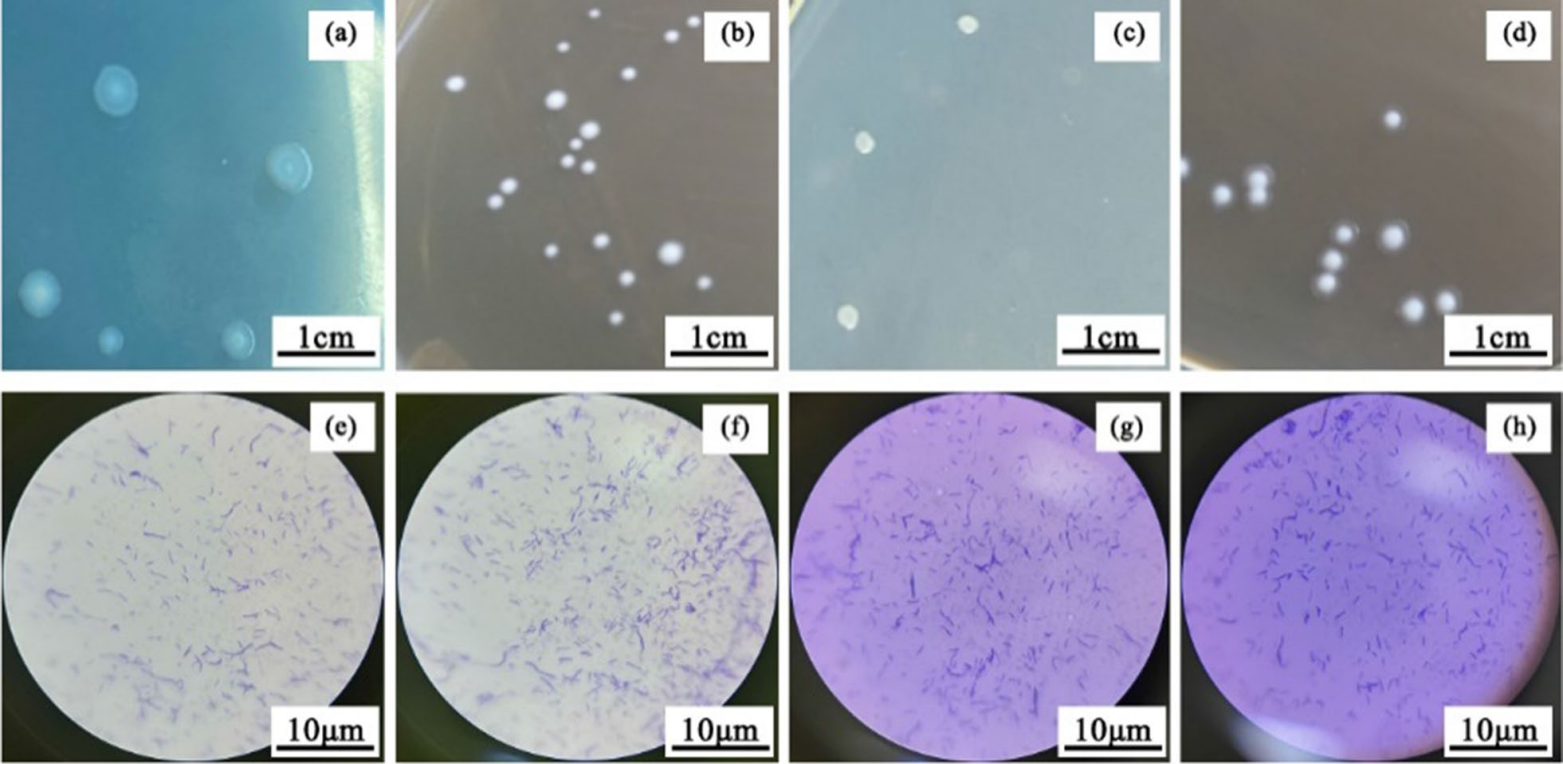
Colonial morphology and microscopic structure of the strain ZC2-1, ZA-1, ZB-1 and ZC-9. **a**–**d** colonial morphology of ZC2-1, ZA-1, ZB-1 and ZC-9. **e**–**h** microscopic structure of ZC2-1, ZA-1, ZB-1 and ZC-9 observed by microscope with 1000 folds amplification

Polyphasic taxonomic analyses of the strains were conducted based on their phenotypic characteristics and phylogenetic analysis (Fig. 1). Strain ZC2-1 form white opaque circular colonies with a white dot in the center (Fig. 1a), and its cells are short rod shape of 0.4–0.8 µm wide by 2.5–4.0 long (Fig. 1e). Strain ZA-1 and ZC-9 both from light milky white small round colonies with moist surface and viscous texture (Fig. 1b, d), except that there is a light white transparent circle around the single colony of ZC-9. Their cells have rod shape of 0.5–1.5 µm wide by 5.0–8.3 µm long (Fig. 1f) and 0.4–0.8 µm wide by 1.8–3.2 µm long (Fig. 1h), respectively. The colony of ZB-1 is dark white colony with irregular edge, rough surface, and viscous texture (Fig. 1c), and its cells show slender rod shape with the size of 0.4–0.8 µm wide by 1.8–3.2 µm long (Fig. 1g).

16S rRNA sequences of the four *B. coagulans* ZC2-1, ZA-1, ZB-1, ZC-9 (shown in Additional file 1) were deposited in GenBank with the accession numbers MW195020, MW504830, MW504831, MW504832, respectively. Among them, strain *B. coagulans* ZC2-1 has been stored in China General Microbiological Culture Collection Center with the preservation numbers of CGMCC No. 22951.

**Fig. 2:**
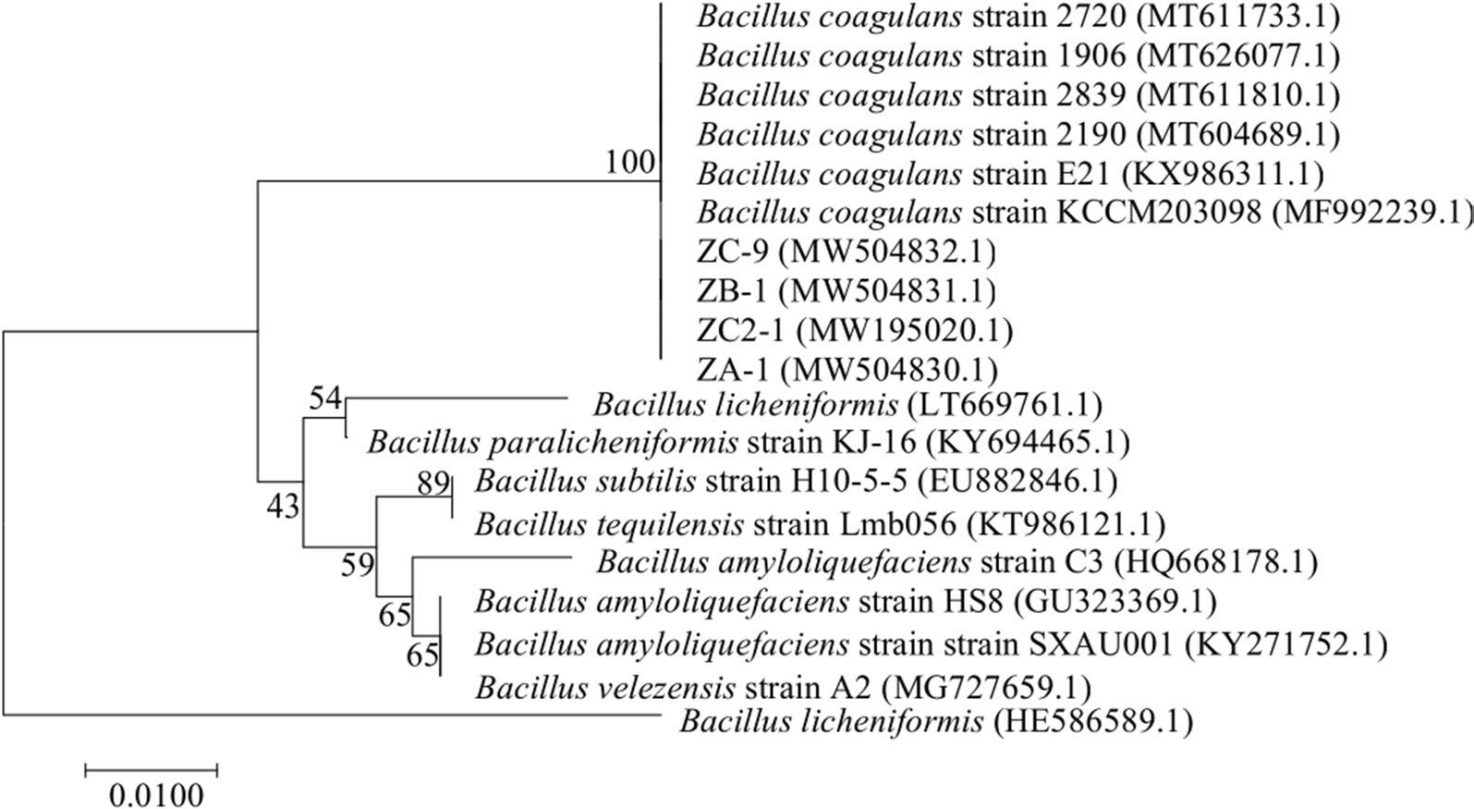
Phylogenetic trees constructed by the Neighbor-Joining approach. The GenBank accession numbers of the strains are shown in the parentheses

The 16S rRNA sequence of ZC2-1 exhibited 99.93% identity with a *B. coagulans* strain (MT604689.1). The 16S rRNA sequence of ZA-1 and ZB-1 revealed 100% identity with the *B. coagulans* strain MT611810.1 and MT611733.1, respectively. And the 16S rRNA sequence of ZC-9 has a 99.93% identity with the *B. coagulans* strain MT626077.1. ZC2-1, ZA-1, ZB-1, ZC-9 was located in the same *B. coagulans* clade (Fig. 2), and their closest relative were *B. coagulans* strain KCCM203098.

### Establishment of coculture system

The four *B. coagulans* strains ZC2-1, ZA-1, ZB-1, ZC-9 were co-fermented with *C. butyricum* DL-1 under the condition described before. After a 24 h culture, the *C. butyricum* spore concentration of four co-culture system were 5.5 × 10^5^, 4.5 × 10^5^, 4.8 × 10^5^, 5.1 × 10^5^ cfu/mL, respectively. The highest *C. butyricum* spore concentration was obtained in coculture system composed of *B. coagulans* ZC2-1 and *C. butyricum* DL-1. Therefore, this co-culture system was studied further.

### The growth curve and pH curve of *C. butyricum *DL-1 and *B. coagulans* ZC2-1

The lag period of *C. butyricum* DL-1 lasted only 4 h in the proliferation medium (Additional file 1: Fig. S1). Then the logarithmic phase began with the cell multiplying rapidly and pH dropping sharply. The stable period began at 12 h, and fermentation process entered the decay period at 20 h with pH value increasing continuously.

*B. coagulans* ZC2-1 grew slowly in the proliferation medium in the lag period with pH decreasing slowly (Additional file 1: Fig. S2). From the 8th hour, the bacteria rapidly propagated into the logarithmic phase and reached the plateau at 16 h.

There is a markedly negative correlation between the changing trend of cell concentration and pH value. In the stable and decay period, bacterial growth was inhibited by low pH, low nutrients concentration and high harmful metabolites concentration caused by bacterial growth. The increasing pH value in the decay period may be related to the autolysis of the bacteria cells. At late logarithmic phase, the bacterial cell concentration reached the highest, and the cells exhibited the highest viability and fertility in the same time. Therefore, the culture broth of *C. butyricum* DL-1 harvested after cultured for 16 h, 12 h for *B. coagulans* ZC2-1, was used as inoculum for co-fermentation process.

### Optimization of mixed fermentation medium composition

It was found that the concentration of *B. coagulans* cells in co-fermentation broth is far inferior to the culture result in its purebred fermentation. Additionally, *B. coagulans* spore yield in co-fermentation process is almost negligible. Therefore, the viable bacteria concentration and spore yield of *C. butyricum* viable were used as medium optimization criterion in this study.

### Effect of carbon source types on the viable counts and spore yield of *C. butyricum* DL-1

**Fig. 3:**
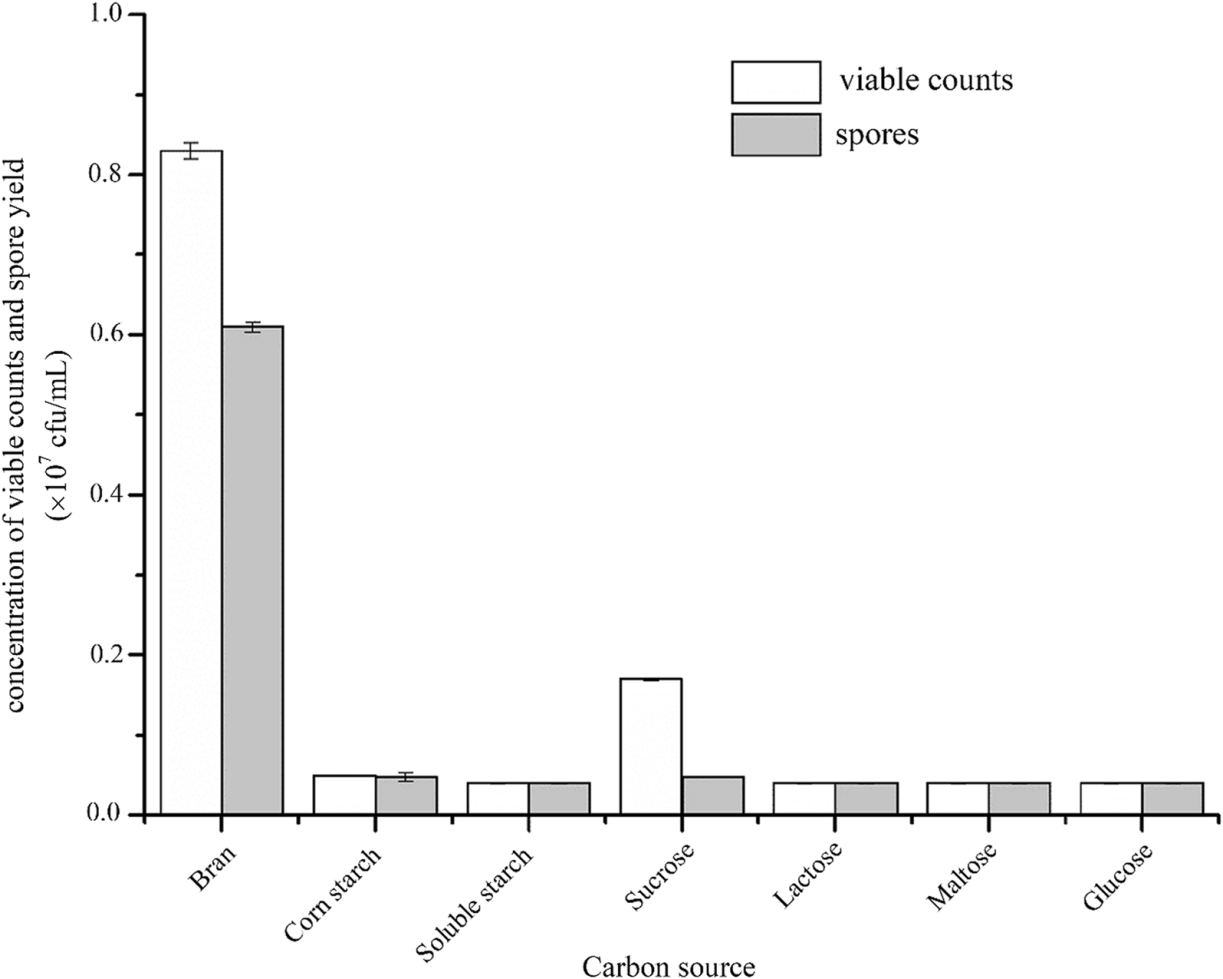
The effect of carbon source types on the viable counts and spore yield of *C. butyricum*

When bran is used as carbon source, the concentration of viable bacteria and spores in the culture broth reached the highest (Fig. 3). The viable counts reached 0.83 × 10^7^ cfu/mL, compared with the result of glucose (0.04 × 10^7^ cfu/mL), there is a 20.75 time improvement (Fig. 3). Besides provision carbon source, bran contains microcomponents such as iron, magnesium, sulfur, phursphorus, vitamin A, vitamins C, various amino acids and other grow factors which facilitate bacterial growth (Ritthibut et al. 2020). Therefore, bran showed much better fermentation performance than other carbon sources. It was used as the only carbon source in further study.

### Effect of nitrogen source sorts on the viable counts and spore yield of *C. butyricum*

When corn steep powder was used as nitrogen source, the number of both viable counts and spores were the highest (Additional file 1: Fig. S3), reaching 5.6 × 10^7^ cfu/mL and 3.5 × 10^7^ cfu/mL respectively. Viable counts increased by 140 times and the spore yield increased by 87.5 times. The reason of the remarkable improvement was that the carbon and nitrogen source in the initial medium was unsuitable for *C. butyricum* DL-1. In order to further increase the viable counts and spore yield of *C. butyricum* DL-1, composite nitrogen source with more comprehensive nutrition was studied.

The concentration of viable bacteria and spores of Group 2 was the highest (Table 1), reaching 7.1 × 10^7^ cfu/mL and 5.8 × 10^7^ cfu/mL respectively, the viable bacteria increased by 177.5 times. The spore rate of Group 1 was the highest, but the number of viable counts and spore was lower than that of Group 2.With rich protein, amino acids, vitamins, minerals and trace grow factors, corn steep powder can provide comprehensive nutrient for the growth of microbes (Zeng et al. 2018).Peptone contains vitamins and other growth factors (Setiari et al. 2016).They are both good choices for bacterial nitrogen source.

**Table 1:** The optimized results of compound nitrogen source

Nitrogen source	*C. butyricum* viable counts (× 10^7^ cfu/mL)	*C. butyricum* spores (× 10^7^ cfu/mL)	Spore rate (%)
Group1 Group2 Group3 Group4 Group5 Group6 Group7 Group8 Group9 Contrast	4.8 7.1 3.1 4.0 5.0 1.8 4.5 4.2 2.3 5.6	4.1 5.8 2.4 2.2 1.7 0.9 2.1 2.7 1.3 3.5	85.4 81.7 77.4 55.0 34.0 50.0 46.7 64.3 56.5 62.5

Group1 to 9 indicate the combination of corn steep powder with soybean meal powder, peptone, beef paste, fishmeal, tryptone, yeast extract, yeast, NH_4_Cl and (NH_4_)_2_SO_4_ respectively. The ratio of corn steep powder to other nitrogen sources is 1:1, and the total nitrogen source content is 15 g/L. 15 g/L corn steep powder was used as control

### Effect of the carbon and nitrogen source concentration on the viable counts and spore yield of *C. butyricum* DL-1

A L_9_ (3^3^) orthogonal table (Table 2) was designed to optimize the concentration of carbon source and nitrogen source. Bran, peptone and corn steep powder concentration were set as factors in this orthogonal test.

**Table 2:** The orthogonal experiment design table (L_9_ (3^3^) for carbon source and nitrogen source concentration and results analysis

Level	Bran (g/L)	Peptone (g/L)	Corn steep powder (g/L)	*C. butyricum* viable counts (× 10^7^ cfu/mL)	*C. butyricum* spores (× 10^7^ cfu/mL)
1	20	15	5	5.8	4.4
2	10	10	15	8.5	7.4
3	20	5	15	6.1	5.2
4	10	15	10	7.4	5.7
5	15	15	15	8.2	6.1
6	20	10	10	5.6	3.9
7	15	10	5	6.5	3.5
8	15	5	10	3.1	2.7
9	10	5	5	5.8	3.5
‾K_1a_	7.23	5.00	6.03		
‾K_2a_	5.93	6.87	5.37		
‾K_3a_	5.83	7.13	7.60		
R_A_	1.40	2.13	2.23		
‾K_1b_	5.53	3.80	3.80		
‾K_2b_	4.10	4..93	4.10		
‾K_3b_	4.50	5.40	6.23		
R_B_	1.43	1.60	2.43		

K value stands for the mean value, for example, K1a, K2a and K3a correspond to A are the mean values of factor A, at level 1, level 2, and level 3, respectively, and so on. R stand for range value, correspond to the different between the highest k value and the lowest k value. English letter A and B in rang analysis results stand for the results of *C. butyricum* DL-1 viable counts and spore yields, respectively

According to the range analysis results (Table 2), the three factors had the similar influence on the viable counts and spore yield of *C. butyricum* DL-1. The order of importance was corn steep powder concentration > peptone concentration > bran concentration. The optimum contents of carbon source and nitrogen source were determined as 10 g/L bran, 15 g/L peptone, and 15 g/L corn steep powder. The verification results conducted under the optimal condition combination were as follows: the viable counts and spore yield of *C. butyricum* reached 8.8 × 10^7^ and 7.6 × 10^7^ cfu/mL, respectively. The number of viable bacteria increased 220 times. The results were better than all those shown in the orthogonal table, which further verified the conclusion drawn by the orthogonal experiment.

### Effect of inorganic salts on the viable counts and spore yield of *C. butyricum* DL-1

Inorganic salts play an important role in the growth of microorganisms. Phosphorus and sulfur element are important component of DNA, RNA and protein. Many metal ions serve as cofactors of metabolic enzymes.

**Fig. 4:**
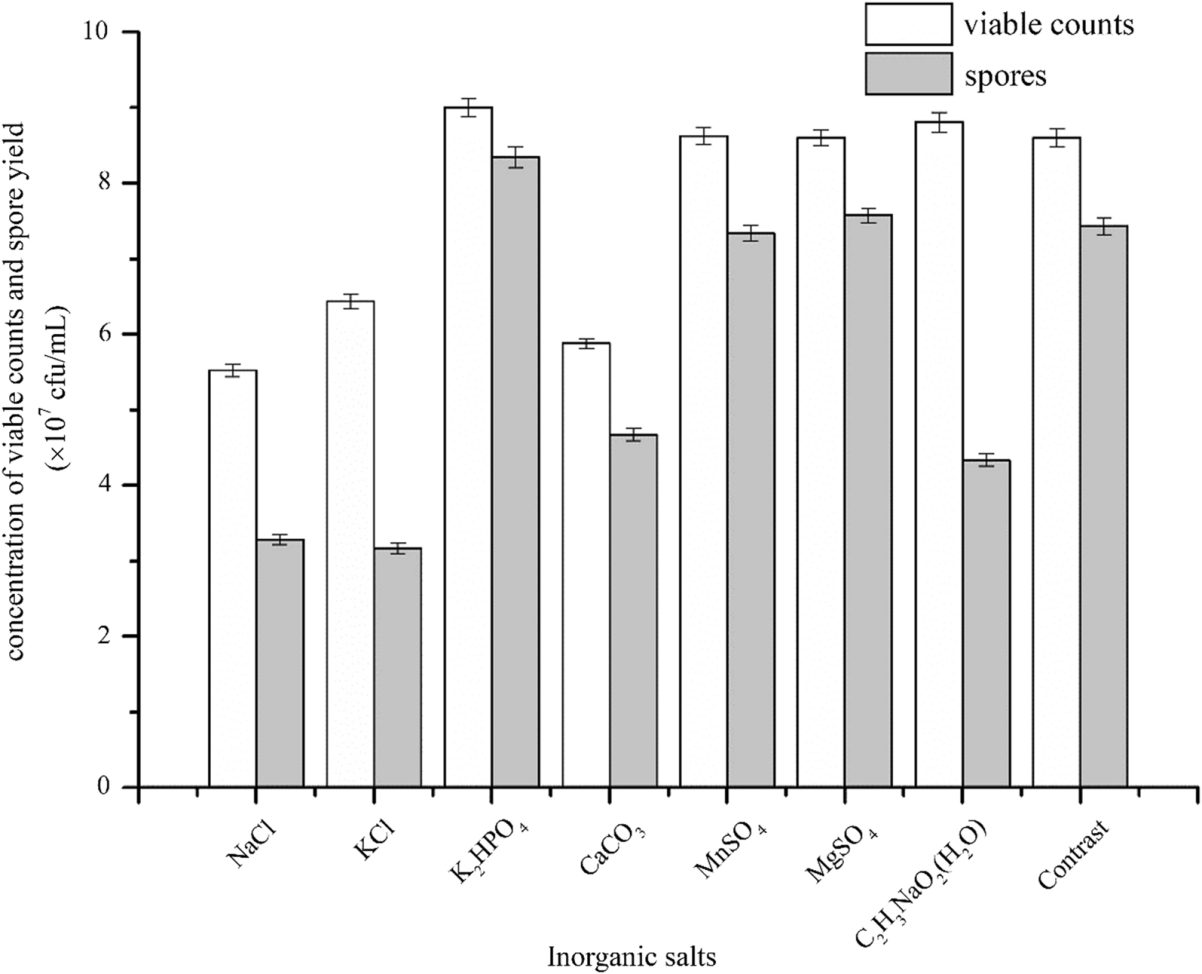
The effect of inorganic salts on *C. butyricum’s* viable counts and spore yield. The concentration of NaCl and KCl was 5 g/L, the concentration of K_2_HPO_4_ and CaCO_3_ was 1 g/L, the concentration of MnSO_4_ and MgSO_4_ was 0.3 g/L, the concentration of Sodium acetate trihydrate was 3 g/L, with no inorganic saltsadded in control experiment

K_2_HPO_4_ was proved to be the top factor in promoting *C. butyricum’*s growth and sporulation (Fig. 4). Sodium acetate trihydrate, MnSO_4_, and MgSO_4_ also exhibited marked enhancement effect, so the combination of K_2_HPO_4_ and the three inorganic salts was further studied. The results showed that the optimal inorganic salts combination for the of coculture of *C. butyricum*DL-1 and *B. coagulans* ZC2-1 was K_2_HPO_4_ and MnSO_4_ (Additional file 1: Table S1), with the viable counts and spores rate reaching 1.04 × 10^8^ cfu/mL and 91.3%, respectively. The concentration of K_2_HPO_4_ and MnSO_4_ was optimized by a L_9_(3^2^) orthogonal experiment (Additional file 1: Tables S2, S3), with K_2_HPO_4_ (A) and MnSO_4_ (B) content as factors.

According to the range analysis results (Additional file 1: Table S3), K_2_HPO_4_ is the most important influence factor for the viable bacteria and spore rate of *C. butyricum* DL-1. The optimum inorganic salt combination was 1 g/L K_2_HPO_4_ and 0.5 g/L MnSO_4_. Under this condition, the viable counts and spore rate of *C. butyricum* DL-1 reached 1.3 × 10^8^ cfu/mL and 92.3%, respectively. The number of viable bacteria increased 325 times.

### Fermentation result in the optimal medium

**Fig. 5:**
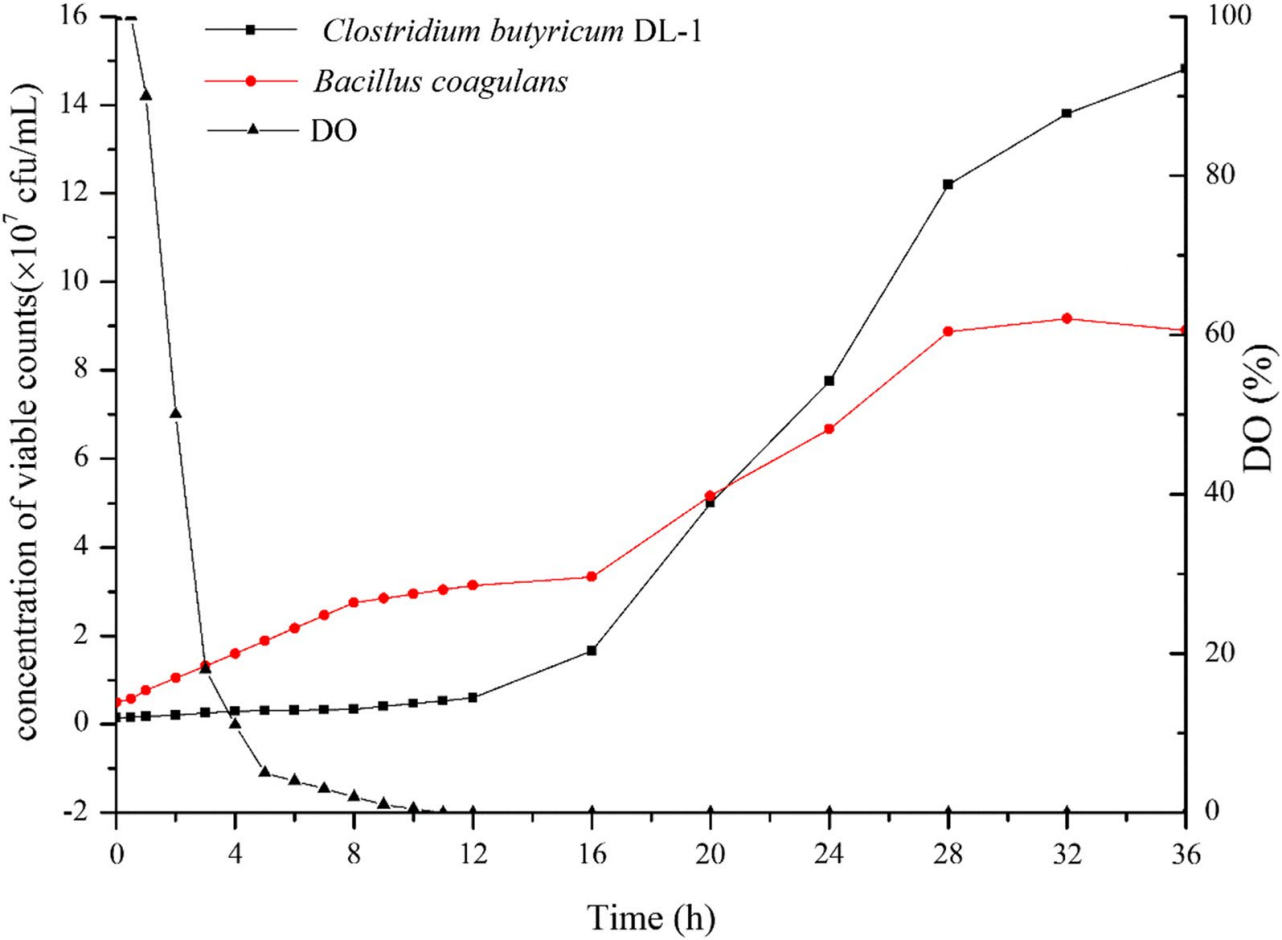
Growth curve and DO curve of the two strains in optimized coculture medium

Different from their growth curves in pure culture, *B. coagulans* ZC2-1 started growth earlier than *C. butyricum* DL-1 in the coculture system. At lag phase, the cells of *B. coagulans* strain ZC2-1 grow gradually from 1 to 2 h (Fig. 5), while Dissolved oxygen (DO) decreased slowly. Then, *B. coagulans* strain ZC2-1 growth enter logarithmic phase, DO decreased rapidly to zero at 10 h, accompanied by the rapid cell proliferation of *B. coagulans* strain ZC2-1. *C. butyricum* DL-1 began to grow at 12 h under the absolute anaerobic condition provided by *B. coagulans* strain, and enter logarithmic phase at 16 h. After a temporary rest, *B. coagulans* strain grew fast at a much bigger speed, which may be caused by the supplement of nutrition. The stable phase of *B. coagulans* strain ZC2-1 and *C. butyricum* DL-1 began at 28 h and 36 h, respectively. At 36 h, the viable counts and spores yield of *C. butyricum* DL-1 both reached the peak value of 1.5 × 10^8^ cfu/mL and 1.4 × 10^8^ cfu/mL respectively. The number of viable bacteria increased 375 times.

## Discussion

Although antibiotic has made significant contribution in protecting human and animal health. Its abuse may lead to environmental antibiotic residues (Silvia Munoz-Price et al. 2016). Residual antibiotics could interfere natural beneficial gut flora and improve pathogenic bacterial antibiotic tolerance (Chen et al. 2019). It could also lead to the spread of antibiotic resistance genes (ARGs) in environment and cause serious health problems by changing human and animals' gut microbiota structure (Ben et al. 2019; Duan et al. 2020).Therefore, The development of ecofriendly disease preventative approaches will be beneficial to the health management in animals farming (Alagawany et al. 2018). Probiotic preparations will not cause drug-resistance. Therefore, they are considered to be the most promising antibiotic substitutes (Ouwehand et al. 2016). Besides feed supplement, probiotics are also used in medicines and healthcare products. Probiotic preparation on the market includes bacteria, fungus and yeast. The commonly used probiotics are the strains of *B. subtilis*, *Lactobacillus* sp., *Bifidobacterium* sp., and *Streptococcus* sp. As an emerging spore forming probiotic, *C. butyricum* does not have big market share yet. So it is of great interest to research simple and cost-effective fermentation process so as to increase its production and application.

Scientists have made great effort to improve the viable counts and spore yield of *C. butyricum* strains. Kong et al. (2004) increased *C. butyricum’s* viable counts by optimizing the medium composition. Li et al. (2020b) reported that pH value was a crucial factor for the spore formation of *C. butyricum*. Down-regulation pH value from 6.5 to 5.5 during the fermentation process promoted *C. butyricum’*s sporulation rate to 90%. For most *C. butyricum*, it is obligatory to provide anaerobic agents or nitrogen gas protection to create an anaerobic fermentation environment, which increase the fermentation equipment investment and operation cost greatly. As an alternative, we established a coculture process of *C. butyricum* DL-1 and *B. coagulans* ZC2-1. In the co-fermentation system, the facultative anaerobic *B. coagulans* strain consumes oxygen in the culture medium and provides anaerobic environment for the strict anaerobic *C. butyricum* DL-1.

Fermentation medium component such as carbon sources, nitrogen sources, inorganic salts and growth factors are important factors affecting microbial growth. Therefore, we optimized the culture medium composition of the co-fermentation process so as to obtain high viable counts and spore yield of *C. butyricum* at low medium cost. Despite the differences among the medium compositions and their concentrations, the cost of reported media for *C. butyricum* cultivation was quite high (He et al. 2017; Li et al. 2019a). In this study, a low-cost medium formula for the co-fermentation of *C. butyricum* DL-1and *B. coagulans* ZC2-1was designed. Readily available and inexpensive food industry byproducts were used as the main raw material so as to further decrease the cost. And a 64.6% reduction in culture medium cost is achieved compared with Qing Kong’ medium (Table 3).

**Table 3:** Cost comparison of our medium and reported medium

Input	Price	Reported Medium^Q^			Newly designed medium	
	(USD/Kg^a^)	(g/L^b^)		(USD/L^c^)	(g/L)	(USD/L)
Glucose	249	2.44		0.608	–	–
Yeast extract	224	2.08		0.466	–	–
Tryptone	448	1		0.045	–	–
(NH_4_)_2_SO_4_	175	0.1		0.018	–	–
NaHCO_3_	142	0.1		0.014	–	–
MnSO_4_·H_2_O	151	0.02		0.003	0.5	0.0755
MgSO_4_·7H_2_O	75	0.02		0.0015	–	–
CaCl_2_	161	0.002		0.0003	–	–
Bran	0.255	–			10	0.00255
Corn steep powder	0.413	–			15	0.006195
Peptone	0.295	–			15	0.004425
K_2_HPO_4_	320	–			1	0.32
Total (USD/L)		1.1558			0.40867	

Q: Formula reported by Qing Kong et al

^a^Cost of each component was calculated based on Sigma-Aldrich prices (accessed on December 2020)

^b^Amount of component (g) per liter of medium

^c^Cost of component per liter of medium

The mixed fermentation of microorganisms with different growing characteristics may solve some intractable problems faced by purebred fermentation. Co-fermentation together with facultative anaerobic strains provides an effective solution for absolute anaerobic bacteria culture. Microbial co-fermentation is widely used in the production of animal feed (da Silva Brito et al. 2020), protein (Jia et al. 2018), drugs (Pettit 2009), foods (Capece et al. 2018), biological control (Ma et al. 2020), and environmental management (Chen et al. 2019). B_12_ was produced by mixed fermentation of *Propionibacterium freudenreichii* and *L. brevis* (Xie et al. 2019); Dairy products (Kongo et al. 2006) and lactic acid (Zhang and Vadlani 2015) was produced by co-fermentation of *B. animalis* and *L. acidophilus*. Cong et al. (2019) used the mixed fermentation broth of *R. nigricans* and *Trichoderma pseudokoningii* to control cucumber wilt, and found that the combined fermentation has a synergistic effect on the control of *Fusarium oxysporum*. Based on the characteristics of *C. butyricum* and *B. coagulans*, a green and energy-saving co-fermentation process was established in this study. Without the need of anaerobic environment, *C. butyricum* co-fermentation process was simplified, and the production cost was reduced greatly.

In the mixed culture of multiple strains, the interaction among the strains should be explored based on their growth characteristics. Viable counts, spore transformation rate, fermentation period and other factors should be considered so as to obtain the maximum benefits. Furthermore, it is necessary to further optimize the co-fermentation process of *C. butyricum* and *B. coagulans* so as to provide theoretical basis and technical guidance for the production of *C. butyricum.*

In this study, the effect of carbon source, nitrogen source and inorganic salts were studied. The co-fermentation medium formula of *C. butyricum *DL-1 and *B. coagulans* ZC2-1was optimized as 10 g/L bran, 15 g/L corn steep powder, 15 g/L peptone, 1 g/L K_2_HPO_4_, and 0.5 g/L MnSO_4_ at pH 7.0. Cultured in the optimized medium formula, the concentration of viable bacteria and spores of *C. butyricum* DL-1 reached 1.5 × 10^8^ cfu/mL and 92.6% after a 36 h static culture at 37 °C. The number of viable bacteria increased 375 times. Besides, the economic assessment revealed the great potential of the medium for *C. butyricum* large-scale production. The co-fermentation process established in this study also provides an effective alternative for the industrial production of other absolute anerobic bacteria.

## Supplementary Information

**Additional file 1.**Sequencing results of 16S rRNA of four *B. coagulans*; **Table S1** The optimized results of composite inorganic salts; **Table S2** Factors and levels of orthogonal experiment design of composite inorganic salts; **Table S3** The orthogonal experiment design table (L9 (32)) and results analysis of composite; **Fig. S1** Growth characteristics and pH change trend of strains *C. butyricum* DL-1; **Fig. S2** Growth characteristics and pH change trend of strains *B. coagulans* ZC2-1; **Fig. S3** The effect of nitrogen source sorts on the viable counts and spore yield of *C. butyricum*.

## Data Availability

All data generated or analyzed during this study are included in this published article and its Additional files.
