# Supplementary material for: Optimization of an economical medium composition for the coculture of Clostridium butyricum and Bacillus coagulans
Source: AMB Express. 2022 Feb 15;12:19. doi: 10.1186/s13568-022-01354-5 (PMC8847521; doi:10.1186/s13568-022-01354-5)
Supplement: Supplementary file 1 — Additional file 1.Sequencing results of 16S rRNA of four B. coagulans; Table S1 The optimized results of composite inorganic salts; Table S2 Factors and levels of orthogonal experiment design of composite inorganic salts; Table S3 The orthogonal experiment design table (L9 (32)) and results analysis of composite; Fig. S1 Growth characteristics and pH change trend of strains C. butyricum DL-1; Fig. S2 Growth characteristics and pH change trend of strains B. coagulans ZC2-1; Fig. S3 The effect of nitrogen source sorts on the viable counts and spore yield of C. butyricum. [file 13568_2022_1354_MOESM1_ESM.docx]

**Optimization of an economical medium composition for the coculture** **of *Clostridium butyricum* and *Bacillus coagulans***

Yonghong Li^1,2^, Yun Wang^1^, Yingying Liu^1^, Xuan Li^1^, Lifei Feng^3^, Keke Li^3*^

**1** Key Laboratory of Advanced Drug Preparation Technologies, Ministry of Education, School of Pharmaceutical Sciences, Zhengzhou University, Henan, Zhengzhou 450001, China

**2** Henan Province Collaborative Innovation Center of New Drug Research and Safety Evaluation, Henan, Zhengzhou 450001, China

**3** HeNan JinBaiHe Biotechnology Co., LTD，Henan, Anyang 450000, China

**Supplementary Material**

Number of:

Pages: 11

Figures: 3

Tables: 3

E-mail address: [likeke202201@163.com](mailto:likeke202201@163.com) (Keke Li)

*Corresponding author:

Keke Li

Tel:+8613203838888

**Sequencing results of 16S rRNA of four *B. coagulans***

***B. coagulans* ZC2-1:**

TGCAGTCGTGCGGACCTTTTAAAAGCTTGCTTTTAAAAGGTTAGCGGCGGACGGGTGAGTAACACGTGGGCAACCTGCCTGTAAGATCGGGATAACGCCGGGAAACCGGGGCTAATACCGGATAGTTTTTTCCTCCGCATGGAGGAAAAAGGAAAGACGGCTTTTGCTGTCACTTACAGATGGGCCCGCGGCGCATTAGCTAGTTGGTGGGGTAACGGCTCACCAAGGCAACGATGCGTAGCCGACCTGAGAGGGTGATCGGCCACATTGGGACTGAGACACGGCCCAAACTCCTACGGGAGGCAGCAGTAGGGAATCTTCCGCAATGGACGAAAGTCTGACGGAGCAACGCCGCGTGAGTGAAGAAGGCCTTCGGGTCGTAAAACTCTGTTGCCGGGGAAGAACAAGTGCCGTTCGAACAGGGCGGCGCCTTGACGGTACCCGGCCAGAAAGCCACGGCTAACTACGTGCCAGCAGCCGCGGTAATACGTAGGTGGCAAGCGTTGTCCGGAATTATTGGGCGTAAAGCGCGCGCAGGCGGCTTCTTAAGTCTGATGTGAAATCTTGCGGCTCAACCGCAAGCGGTCATTGGAAACTGGGAGGCTTGAGTGCAGAAGAGGAGAGTGGAATTCCACGTGTAGCGGTGAAATGCGTAGAGATGTGGAGGAACACCAGTGGCGAAGGCGGCTCTCTGGTCTGTAACTGACGCTGAGGCGCGAAAGCGTGGGGAGCAACAGGATTAGATACCCTGGTAGTCCACGCCGTAAACGATGAGTGCTAAGTGTTAGAGGGTTTCCGCCCTTTAGTGCTGCAGCTAACGCATTAAGCACTCCGCCTGGGGAGTACGGCCGCAAGGCTGAAACTCAAAGGAATTGACGGGGGCCCGCACAAGCGGTGGAGCATGTGGTTTAATTCGAAGCAACGCGAAGAACCTTACCAGGTCTTGACATCCTCTGACCTCCCTGGAGACAGGGCCTTCCCCTTCGGGGGACAGAGTGACAGGTGGTGCATGGTTGTCGTCAGCTCGTGTCGTGAGATGTTGGGTTAAGTCCCGCAACGAGCGCAACCCTTGACCTTAGTTGCCAGCATTCAGTTGGGCACTCTAAGGTGACTGCCGGTGACAAACCGGAGGAAGGTGGGGATGACGTCAAATCATCATGCCCCTTATGACCTGGGCTACACACGTGCTACAATGGATGGTACAAAGGGCTGCGAGACCGCGAGGTTAAGCCAATCCCAGAAAACCATTCCCAGTTCGGATTGCAGGCTGCAACCCGCCTGCATGAAGCCGGAATCGCTAGTAATCGCGGATCAGCATGCCGCGGTGAATACGTTCCCGGGCCTTGTACACACCGCCCGTCACACCACGAGAGTTTGTAACACCCGAAGTCGGTGAGGTAACCTTACGGAGCCAGCCGCCGAAGTGAC

***B. coagulans* ZA-1:**

ACCTGCCTGTAAGATCGGGATAACGCCGGGAAACCGGGGCTAATACCGGATAGTTTTTTCCTCCGCATGGAGGAAAAAGGAAAGACGGCTTCTGCTGTCACTTACAGATGGGCCCGCGGCGCATTAGCTAGTTGGTGGGGTAACGGCTCACCAAGGCAACGATGCGTAGCCGACCTGAGAGGGTGATCGGCCACATTGGGACTGAGACACGGCCCAAACTCCTACGGGAGGCAGCAGTAGGGAATCTTCCGCAATGGACGAAAGTCTGACGGAGCAACGCCGCGTGAGTGAAGAAGGCCTTCGGGTCGTAAAACTCTGTTGCCGGGGAAGAACAAGTGCCGTTCGAACAGGGCGGCGCCTTGACGGTACCCGGCCAGAAAGCCACGGCTAACTACGTGCCAGCAGCCGCGGTAATACGTAGGTGGCAAGCGTTGTCCGGAATTATTGGGCGTAAAGCGCGCGCAGGCGGCTTCTTAAGTCTGATGTGAAATCTTGCGGCTCAACCGCAAGCGGTCATTGGAAACTGGGAGGCTTGAGTGCAGAAGAGGAGAGTGGAATTCCACGTGTAGCGGTGAAATGCGTAGAGATGTGGAGGAACACCAGTGGCGAAGGCGGCTCTCTGGTCTGTAACTGACGCTGAGGCGCGAAAGCGTGGGGAGCAAACAGGATTAGATACCCTG

***B. coagulans* ZB-1:**

TGCCTGTAAGATCGGGATAACGCCGGGAAACCGGGGCTAATACCGGATAGTTTTTTCCTCCGCATGGAGGAAAAAGGAAAGACGGCTTCTGCTGTCACTTACAGATGGGCCCGCGGCGCATTAGCTAGTTGGTGGGGTAACGGCTCACCAAGGCAACGATGCGTAGCCGACCTGAGAGGGTGATCGGCCACATTGGGACTGAGACACGGCCCAAACTCCTACGGGAGGCAGCAGTAGGGAATCTTCCGCAATGGACGAAAGTCTGACGGAGCAACGCCGCGTGAGTGAAGAAGGCCTTCGGGTCGTAAAACTCTGTTGCCGGGGAAGAACAAGTGCCGTTCGAACAGGGCGGCGCCTTGACGGTACCCGGCCAGAAAGCCACGGCTAACTACGTGCCAGCAGCCGCGGTAATACGTAGGTGGCAAGCGTTGTCCGGAATTATTGGGCGTAAAGCGCGCGCAGGCGGCTTCTTAAGTCTGATGTGAAATCTTGCGGCTCAACCGCAAGCGGTCATTGGAAACTGGGAGGCTTGAGTGCAGAAGAGGAGAGTGGAATTCCACGTGTAGCGGTGAAATGCGTAGAGATGTGGAGGAACACCAGTGGCGAAGGCGGCTCTCTGGTCTGTAACTGACGCTGAGGC

***B. coagulans* ZC-9:**

TGCCTGTAAGATCGGGATAACGCCGGGAAACCGGGGCTAATACCGGATAGTTTTTTCCTCCGCATGGAGGAAAAAGGAAAGACGGCTTTTGCTGTCACTTACAGATGGGCCCGCGGCGCATTAGCTAGTTGGTGGGGTAACGGCTCACCAAGGCAACGATGCGTAGCCGACCTGAGAGGGTGATCGGCCACATTGGGACTGAGACACGGCCCAAACTCCTACGGGAGGCAGCAGTAGGGAATCTTCCGCAATGGACGAAAGTCTGACGGAGCAACGCCGCGTGAGTGAAGAAGGCCTTCGGGTCGTAAAACTCTGTTGCCGGGGAAGAACAAGTGCCGTTCGAACAGGGCGGCGCCTTGACGGTACCCGGCCAGAAAGCCACGGCTAACTACGTGCCAGCAGCCGCGGTAATACGTAGGTGGCAAGCGTTGTCCGGAATTATTGGGCGTAAAGCGCGCGCAGGCGGCTTCTTAAGTCTGATGTGAAATCTTGCGGCTCAACCGCAAGCGGTCATTGGAAACTGGGAGGCTTGAGTGCAGAAGAGGAGAGTGGAATTCCACGTGTAGCGGTGAAATGCGTAGAGATGTGGAGGAACACCAGTGGCGAAGGCGGCTCTCTGGTCTGTAACTGACGCTGAGGCGCGAAAGCGTGGGGAGCAAACAGGATTAGA

**Table S1** The optimized results of composite inorganic salts

| Inorganic salts | *C.butyricum* viable counts | *C. butyricum* spore yield | Spore rate |
| --- | --- | --- | --- |
|  | (×10^7^cfu/mL) | (×10^7^cfu/mL) | (%) |
| Group1 | 8.8 | 7.8 | 88.6 |
| Group2 | 10.4 | 9.5 | 91.3 |
| Group3 | 9.6 | 6.3 | 65.6 |
| Group4 | 9.1 | 8.2 | 90.1 |

Group 1 to Group 3 stand for the combination of K_2_HPO_4_ and MgSO_4_, MnSO_4_ and Sodium acetate trihydrate, respectively. Control is K_2_HPO_4_ alone.

**Table S2** Factors and levels of orthogonal experiment design of composite inorganic salts.

| Level | Factor | |
| --- | --- | --- |
|  | A: K_2_HPO_4_  (g/L) | B: MnSO_4_  (g/L) |
| 1  2  3 | 1  2  3 | 0.3  0.4  0.5 |

**Table S3** The orthogonal experiment design table (L_9_ (3^2^)) and results analysis of composite inorganic salts.

| Level | | K_2_HPO_4_  (g/L) | | MnSO_4_  (g/L) | *C. butyricum* viable counts  (×10^7^cfu/mL) | *C. butyricum* spores  (×10^7^cfu/mL) |
| --- | --- | --- | --- | --- | --- | --- |
| 1  2  3  4  5  6  7  8  9  ‾K_1c_  ‾K_2c_  ‾K_3c_  R_C_  ‾K_1d_  ‾K_2d_  ‾K_3d_  R_D_ | A1  A3  A2  A2  A1  A2  A1  A3  A3  11.33  7.53  6.60  4.73  10.13  5.47  4.33  5.80 | | B2  B1  B2  B3  B3  B1  B1  B2  B3  7.27  8.20  10.00  2.73  5.67  6.30  7.97  2.30 | | 11.0  4.7  6.5  9.0  13.0  7.1  10.0  7.1  8.0 | 9.3  3.2  5.2  6.5  12.0  4.7  9.1  4.4  5.4 |

K value stands for the mean value, for example, K1c, K2c and K3c correspond to A are the mean values of factor A, at level 1, level 2, and level 3, respectively, and so on. R stand for range value, correspond to the different between the highest k value and the lowest k value. English letter C and D in rang analysis results stand for the results of *C. butyricum* DL-1 viable counts and spore yields, respectively.





**Fig.S1** Growth characteristics and pH change trend of strains *C. butyricum* DL-1





**Fig.S2** Growth characteristics and pH change trend of strains *B. coagulans* ZC2-1





**Fig.S3** The effect of nitrogen source sorts on the viable counts and spore yield of *C. butyricum*
